# Quantitative resistance can lead to evolutionary changes in traits not targeted by the resistance QTLs

**DOI:** 10.1111/eva.12130

**Published:** 2014-01-02

**Authors:** Femke Van den Berg, Christian Lannou, Christopher A Gilligan, Frank van de Bosch

**Affiliations:** 1 Department of Computational and Systems Biology, Rothamsted ResearchHarpenden, Hertfordshire, UK; 2 INRA, UMR 1290 BiogerThiverval Grignon, France; 3 Department of Plant Sciences, University of CambridgeCambridge, UK

**Keywords:** erosion of resistance, life cycle trait adaptation, plant breeding, *Puccinia triticina*, quantitative pathogenicity, quantitative resistance, wheat

## Abstract

This paper addresses the general concern in plant pathology that the introduction of quantitative resistance in the landscape can lead to increased pathogenicity. Hereto, we study the hypothetical case of a quantitative trait loci (QTL) acting on pathogen spore production per unit lesion area. To regain its original fitness, the pathogen can break the QTL, restoring its spore production capacity leading to an increased spore production per lesion. Or alternatively, it can increase its lesion size, also leading to an increased spore production per lesion. A data analysis shows that spore production per lesion (affected by the resistance QTL) and lesion size (not targeted by the QTL) are positively correlated traits, suggesting that a change in magnitude of a trait not targeted by the QTL (lesion size) might indirectly affect the targeted trait (spore production per lesion). Secondly, we model the effect of pathogen adaptation towards increased lesion size and analyse its consequences for spore production per lesion. The model calculations show that when the pathogen is unable to overcome the resistance associated QTL, it may compensate for its reduced fitness by indirect selection for increased pathogenicity on both the resistant and susceptible cultivar, but whereby the QTLs remain effective.

## Introduction

### What are the evolutionary consequences of deploying quantitative resistance?

Cultivar resistance is an efficient, environmentally benign, method of disease control that could allow for a reduction in the use of fungicides in agriculture. Conventional resistance breeding has however mainly focussed on qualitative resistance (Johnson 1992), a form of resistance that is highly efficient but that can, in many cases, easily be overcome by the pathogen. The recurrent deployment of such major resistance genes over large areas has in most cases led to the rapid breaking of resistance and the development of new virulent pathogen strains due to mutation and deletion events (McDonald and Linde 2002; Deacon 2006). Breeders now see quantitative resistance as an alternative approach for developing durably resistant cultivars. Quantitative resistance, although less efficient, is considered more durable than qualitative resistance, mainly because its genetic determinism is more complex. In most cases, quantitative resistance is under the control of multiple genes (Kuo and Wang 2010; González et al. 2012), often referred to as minor-genes, such that the pathogen requires multiple mutations and/or recombinations to overcome the resistance. Adaptation to resistance in this case is expected to result in a gradual erosion of the resistance efficacy, rather than a sudden breakdown (McDonald and Linde 2002; Mundt et al. 2002; for an illustration). Very little is known, however, about the consequences for pathogen evolution of deploying quantitative resistance at a large scale.

### The most intuitive effect: overcoming quantitative trait loci

The most intuitive effect of using a cultivar with quantitative resistance is that the pathogen will in time overcome the resistance mechanisms. Quantitative resistance is usually described by quantitative trait loci (QTLs) having an effect on the pathogen development rate and it is assumed that, like for qualitative resistance, the resistance determined by QTLs can be overcome by the pathogen. Some experimental studies have now been published showing how a pathogen population can adapt to cultivars with quantitative resistance whereby selection leads to the pathogen overcoming the resistance QTLs (see e.g. Lehman and Shaner 1997; Palloix et al. 2009). In this paper, however, we examine a different pathogen adaptation scenario by considering that (i) the different traits that determine quantitative pathogenicity may evolve independently and (ii) that a resistance QTL may specifically affect one of these traits.

### The alternative effect

A plant–pathogen interaction can be described by several well-defined traits, such as the infection efficacy, the latent period or lesion size (Pariaud et al. 2009a; Lannou 2012). There is experimental evidence that lesion size, defined as the area of the spore producing surface (in mm^2^), and spore production capacity, defined as the amount of spores produced per unit lesion area (micrograms of spores/mm^2^), have independent genetic support and can evolve separately in *Puccinia triticina* (Pariaud et al. 2009b; Lannou 2012) and in other plant pathogens (Carlisle et al. 2002). In the host plant, quantitative resistance is sometimes found to affect pathogen development in a pleiotropic way (Lehman and Shaner 1997), but its decomposition into elementary components shows that a QTL may specifically target a single trait of the host–pathogen interaction (Chung et al. 2010). Based on these facts, we hypothesise that, besides overcoming the resistance QTLs, there are other ways for the pathogen to increase its fitness when confronted with a quantitatively resistant cultivar.

### Focus of this paper

We ask the following question: when a quantitative trait is limited by the presence of a QTL in the host plant, can this trait still increase through indirect selection on another trait? In this case, the QTL would remain effective (would not be overcome), but the pathogen would compensate for its effect by indirect selection. More specifically, we consider the case of a QTL acting on spore production capacity (spore production per unit lesion area), resulting in a reduction of the number of spores produced by a lesion, which is an important component of pathogen fitness accounting for the pathogen's transmission capacity. Considering that the spore production per lesion is a composite trait that depends both on the spore production capacity of the infected tissue and on the lesion size (Pariaud et al. 2009b), we assume that restoring the spore production per lesion may be achieved in two ways: either by overcoming the QTL (in which case the spore production per lesion is restored by restoring the spore production capacity of the infected tissue) or by increasing lesion size (in which case the QTL remains effective). When the resistance QTL remains effective, but the pathogen experiences quantitative trait adaptation resulting in larger lesion sizes, this leads to an increased number of spores produced per lesion even though the QTL is not overcome. The latter case is the scenario of interest in this paper.

We will therefore examine whether changes in lesion size can allow the pathogen to regain a high spore production per lesion on resistant plants bearing a QTL that reduces the spore production capacity of the pathogen. We will consider the consequences of such pathogen evolution on the quantitative pathogenicity on resistant plants as well as on susceptible plants. Hereto, we first analyse a set of data and show that the spore production per lesion and lesion size are positively correlated. In a second step, we model the effect of the evolution of the pathogen towards increased lesion size and analyse the consequences of this on the spore production per lesion on both the resistant and the susceptible cultivar.

## Materials and methods

Based on the literature reviewed above, in this paper, we will consider that the amount of spores produced by an individual lesion (resulting from a single pathogen infection) depends both on the lesion size and spore production capacity of the pathogen. It is assumed that the spore production capacity is limited by the presence of quantitative resistance in the host, specifically affecting this trait. We then focus on the capacity of the pathogen to restore its fitness by adapting towards an increased lesion size. Therefore, we will first consider the relationship between spore production per lesion and lesion size (for a fixed spore production capacity), whereby the first part of the paper describes the analysis of a large data set to establish the existence of cultivar-specific relationships between lesion size and spore production per lesion for a wheat pathogen, *Puccinia triticina*. The basidiomycete *Puccinia triticina* (Uredinales) is highly specialised to common wheat and durum wheat and has a worldwide distribution (Bolton et al. 2008). The second part describes how these cultivar-specific relationships are then used in a model study to investigate the adaptation of lesion size for a plant pathogen in the presence of a resistant host cultivar that affects another pathogen trait, that is, the spore production capacity. In this paper, the term lesion denotes a restricted host surface area, such as a leaf rust pustule, that can be colonised by a pathogen individual.

### Experimental procedures

Here, we only give a brief description of the experimental procedures, which are described in full in Pariaud (2008) and Pariaud et al. (2009a,b2009b). Twelve pathogen isolates were tested on five wheat varieties (namely Soissons, Festival, Morocco, Scipion and Thesee), with mostly five replicates per isolate/variety combination [see Table S1 in electronic supplementary material (ESM)]. Experiments were performed in a greenhouse on adult wheat plants, grown under standardised conditions. Each replicate consisted of one pot containing one main wheat stem, of which the flag leaf was inoculated. All inoculations were performed with freshly produced uredospores. The plants were inoculated at the heading or flowering stage by brushing spores on leaf sections of 8 cm in length with a soft brush. During the sporulation period, the leaves were placed in open plastic tubes to collect the spores. Spores were collected at two successive dates around the middle of the pathogen multiplication cycle, transferred into Eppendorf tubes and weighed. Image analysis was used to determine the number of lesions and the sporulating tissue areas. Lesion size (mm^2^) was calculated as the sporulating area divided by the total number of lesions. Spore production per lesion (μg of spores) was calculated as the amount of spores produced between the collection dates divided by the number of lesions and the spore production capacity per lesion (μg of spores per mm^2^ of lesion) was calculated as the amount of spores produced between the collection dates divided by the sporulating areas.

### Data analysis

The above-described data set is used to characterise the relationship between spore production per lesion and lesion size and investigate the effect that host resistance has on this relationship. Cultivars Soissons (*Soi*) and Morocco (*Mor*) are susceptible to all isolates tested, whereas the other cultivars, Festival (*Fes*), Scipion (*Scp*) and Thésee (*The*) are only susceptible to a subset of the isolates (see Table S1 in ESM). There are therefore three classes of isolate–cultivar interactions: (i) *Mor*,*Soi*,*Fes* and *Scp* have a total of six isolates in common; (ii) *Mor*,*Soi* and *The* also have six isolates in common and (iii) *Soi* and *Mor* have all twelve isolates in common.

We used a linear model to test our first hypothesis that there is a significant positive relationship between the spore production per lesion, *Sp*, and the sporulating lesion area (lesion size), *A*, and that this relationship is cultivar specific. The full model is thus given by


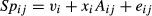
(1)

whereby, *v*_*i*_ represents the intercept of the regression line for cultivar *i*,*x*_i_ represents the slope of the regression line for cultivar *i* and e_*ij*_ represents the residual for the *j*th replicate of the *i*th cultivar.

Our second hypothesis that the isolates lie in a fixed order across the regression lines, which would suggest that adaptation results in a shift along the relationship between lesion size and spore production per lesion, was tested by a Spearman's ranking coefficient test (Sprent 1993) of the ranks of the isolate-means along this relationship for the different host cultivars. The order of the isolates was determined by projecting the isolate-means (according to their perpendicular) onto the cultivar lines as estimated from the linear regression. All statistical tests were performed with the statistical computer package GenStat™ (Payne et al. 2009).

### The epidemic model

The model represents a crop–pathogen system whereby the total host population, expressed in leaf area densities (leaf surface area per m^2^), comprises a susceptible, *H*_*S*_, and a quantitatively resistant, *H*_*R*_, cultivar. A fraction, *θ*, of the fields is planted with the quantitatively resistant cultivar, whilst the remaining fields are planted with the susceptible cultivar. In an agricultural system, this fraction is controlled by farmers. The leaf area densities of the cultivars are assumed to be directly related to their proportions in the landscape. The plants grow with a cultivar-specific intrinsic growth rate *g*_*i*_, with *i *= {*S*(usceptible), *R*(esistant)}, and has a total carrying capacity of *K*. It is convenient here to assume continuous harvest, that is, host plants have a cultivar-specific constant mortality rate *ω*_*i*_. Such a simplification is frequently applied in epidemic models and is assumed acceptable for studying long-term dynamics. The plants in the landscape are affected by a pathogen that can infect both the susceptible and the resistant cultivar, whereby infection by a pathogen spore results in latent tissues *E*_*S*_ and *E*_*R*_ on the susceptible and the resistant cultivar, respectively. The latent tissue becomes infectious after a cultivar-specific latent period of 1/γ_*i*_, resulting in infectious tissues *I*_*S*_ and *I*_*R*_ on the susceptible and the resistant cultivar, respectively. Once infectious, lesions produce a host-cultivar-specific number of spores per unit time, α_*i*_. The total number of spores produced per unit time on cultivar *i* thus depends on the spore production capacity (i.e. spores per mm^2^ lesion surface area) of the pathogen on cultivar *i*, 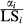, multiplied with the total infectious lesion area of the pathogen on cultivar *i*,*I*_*i*_. Lesions have a cultivar-specific infectious period, 1/*μ*_*i*_ and the infection efficiency of a spore produced by a lesion on cultivar *n* with *n *= {*S*,*R*} that lands on cultivar *m* with *m *= {*S*,*R*} is denoted by *β*_mn_. The full model is given by,


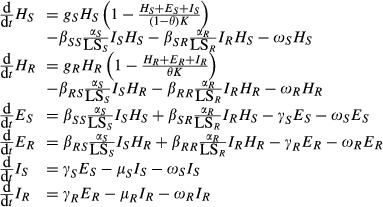
(2)

The model variables and parameters are summarised in Table 1.

**Table 1:** Description of the model variables and parameters including their default values.

Symbol	Description	Default value
*Variables*		
*H*_*i*_	Healthy leaf area density cultivar *i*	
*E*_*i*_	Density of latent tissue area on cultivar *i*	
*I*_*i*_	Density of infectious tissue area on cultivar *i*	
*LS*_*i*_	Lesion size on cultivar *i*	
*α*_*i*_	Spore production capacity, i.e. number of spores produced per lesion per time unit	
*Parameters*		
θ	Fraction of the resistant cultivar within the landscape	[0…1]
*g*_*i*_	Cultivar-specific intrinsic host growth rate	1.5
*K*	Host population total carrying capacity	500
*ω*_*i*_	Cultivar-specific host mortality rate	0.02
1/*γ*_*i*_	Cultivar-specific latent period	5
1/*μ*_*i*_	Cultivar-specific infectious period	10
*β*_*mn*_	Infection efficiency of a spore produced by a lesion on cultivar *n* that lands on cultivar *m*	5 × 10^−6^
*a*	Upper asymptote of the spore production capacity versus lesion size relationship	200
*b*	Lesion size displacement of the spore production capacity versus lesion size relationship	−50
*c*	Slope of the spore production capacity versus lesion size relationship	−10
*ɛ*	Permanent shift in lesion size, i.e. lesions are generally smaller on the resistant cultivar	1500
*σ*	Relative strength of resistance for resistance scenario 1	0.8
*ρ*	Relative strength of resistance for resistance scenario 2	0.8

### Modelling host resistance

Within the data analysis, we test for linear relationships between spore production per lesion (*α*_*i*_ in the model) and lesion size (LS_*i*_ in the model). In practice, this relationship is, however, likely to be asymptotically bounded, because firstly the size of a leaf rust lesion is limited by mechanical or physiological constraints (Lannou 2012) and secondly a biotrophic pathogen such as leaf rust feeds from host tissues surrounding the sporulating area and observations show that very large lesions produce relatively less spores and rapidly show necrosis at their centre (Azzimonti et al. 2013). It would therefore not be logical to assume that spore production increases indefinitely with increased lesion size. This limitation is, however, unlikely to be identified within the data due to the lack of observations for extreme values. So, to avoid negative as well as unrealistically high values, the relationship between spore production per lesion per unit time, *α*_*i*_, and lesion size, LS_*i*_, is modelled by a sigmoid-shaped Gompertz curve (Fig. 3A,B). For lesions developing on the susceptible cultivar, the relationship is given by


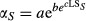
(3)

with *a* the upper asymptote, *b* the lesion size displacement and *c* the slope.

In the model, resistance can affect the spore production per lesion in different ways. For example, host resistance can affect the upper asymptote (resistance scenario 1) or the slope of the relationship (resistance scenario 2). The relationship between spores produced per lesion as a function of lesion size on the resistant cultivar can thus be given by


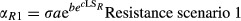
(4)


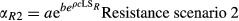
(5)

where *σ* and *ρ* denote the relative strength of resistance with 0 < *σ *< 1 and 0 < *ρ *< 1. Note that low *σ* or *ρ* values both denote a high level of resistance.

We assume that lesion size adaptation occurs in such a manner that a change in lesion size on one cultivar results in an identical lesion size increase or decrease on the other cultivar. Because the presence of quantitative resistance in the host may affect the pathogen pleiotropically (Lehman and Shaner 1997), we include a general resistance penalty in addition to the specific QTL studied, which leads to lesions being generally smaller on the resistant cultivar as compared to the susceptible cultivar. This pleiotropic effect simply adds to the specific effect of the QTL on the spore production capacity. Given the lesion size on the susceptible cultivar, *LS*_*S*_, and eqns (3)–(5), the lesion size on the resistant cultivar can, after some reorganising of the equations, be calculated from


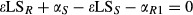
(6)

and


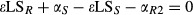
(7)

for resistance scenarios 1 and 2, respectively, whereby *ε* represents the permanent shift in lesion size representing the fact that lesions are generally smaller on the resistant cultivar.

### Pathogen adaptation dynamics

We assume here that the pathogen is not able to overcome the resistance, that is, mutations for increased spore production capacity are not allowed. However, the lesion size is allowed to evolve. We thus determine the optimum lesion sizes on both the susceptible and resistant cultivar [note that they are correlated; see eqns (6) and 7] for different levels of resistance and for different fractions of the resistant cultivar within the landscape. Because the lesion sizes on the resistant cultivar are calculated according to the lesion sizes the pathogen reaches on the susceptible cultivar, the optimal lesion size of a pathogen in a landscape that contains only plants of the resistant cultivar cannot be derived directly. Instead, we use the model equations representing a landscape containing only plants of the susceptible cultivar, that is, *θ *= 0 and then replacing all cultivar-specific parameters with those for the resistant cultivar.

The ‘optimum’ strategy to adopt for a given fraction of resistant host within the landscape and a given level of resistance can be determined by using the method of pairwise invasibility plots (PIP, Geritz et al. (1998); see ESM for mathematical details). In brief: we determine whether a mutant that has a slightly altered lesion size can invade the resident pathogen system when at equilibrium. If this invasion is successful, the mutant phenotype out-competes the resident strain and itself becomes the new resident phenotype. A sequence of invasion and replacement events occurs until the resident population adopting the new strategy cannot be invaded by mutant phenotypes with similar strategies. At this point, an evolutionary endpoint is reached, known as a singular strategy. In this paper, for each parameter combination, we always found a single singular strategy that was both evolutionary and continuously stable, that is, the singular strategy is a continuously stable strategy (CSS; Maynard Smith 1982). This means that evolution is towards the singular strategy and once reached the pathogen population cannot be invaded by mutants with similar strategies.

## Results

### Data analysis results

**Figure 1:**
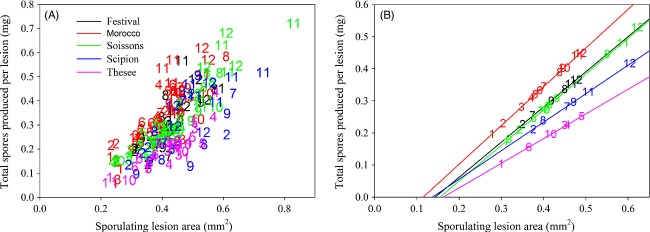
Spore production per lesion (mg) and lesion size relationship for (A) the raw data and (B) the ranking of the transposed means across the estimated cultivar regression lines with the cultivar Morocco as the reference cultivar. The numbers refer to different isolates (see electronic supplementary material for further details).

The regression analysis of the raw data on spore production per lesion (mg) and lesion size relationship as presented in Fig. 1A established that there is a clear monotonically rising relationship between lesion size and spore production per lesion for *P. triticina* on wheat (*P *<* *0.001). Moreover, the regression lines for the individual cultivars have significantly different intercepts (*P *<* *0.001) and slopes (*P *=* *0.044), indicating that on some cultivars the pathogen strains generally have a reduced spore production per lesion (see ESM for detailed regression analysis results). This host effect can be interpreted as a consequence of the resistance factors affecting the spore production capacity. The relationship thus reveals that spore production per lesion and lesion size are dependent quantitative traits linked through a positive relationship.

The cultivar-specific regression lines are shown graphically in Fig. 1. Projection (according to their perpendicular) of the mean isolate values onto the regression lines gives a graphical representation of the order of the isolates across the cultivar-specific relationships. For each of the three isolate–cultivar interaction classes (*cf*. Table S1 in ESM), a Spearman's ranking correlation coefficient test revealed that, for all cultivars except *The*, the order of the isolate-means is significantly correlated (*ρ *> 0.829) for all significant correlations) across the different cultivars. Ranking according to isolate medians rather than means resulted in a slight change in the order of the isolates across the cultivars, but these differences were not significant (see ESM for detailed results on both spearman ranking correlation analyses).

### Model analysis results

**Figure 2:**
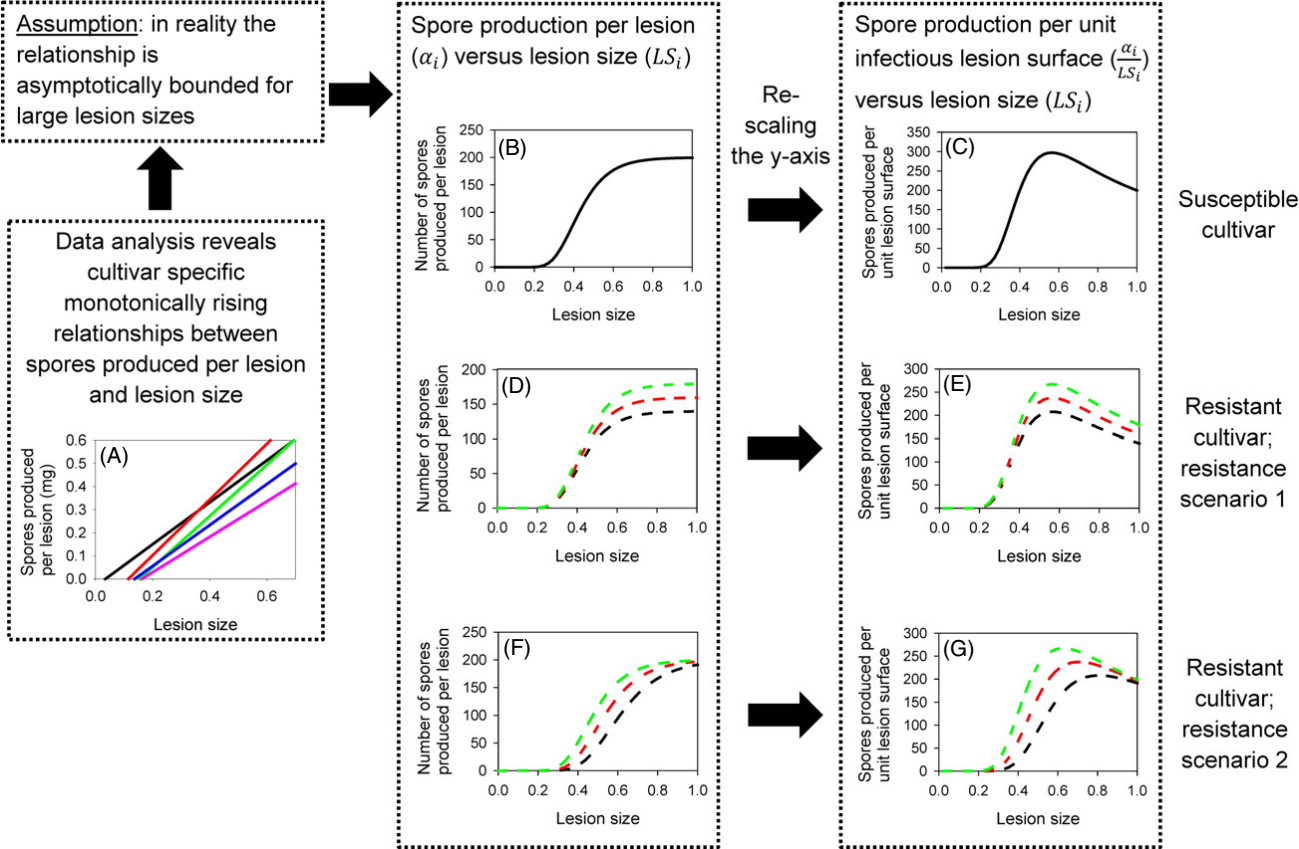
Graphical representation of how the relationship between the spore production per lesion in mg as a function of lesion size as found by the data analysis [see (A)] is translated into a relationship between spore production per unit infectious lesion area versus lesion size [see (C), (E) and (G)]. This shows that in homogeneous landscapes containing a single cultivar, the pathogens have a clear optimum lesion size. Note however that these graphs are representative for lesions on plants within a homogeneous landscape containing a single cultivar only. When both cultivars are present in the landscape, pathogen adaptation is not necessarily towards these optima (see main text). The changes from (B), (D) and (F) to (C), (D) and (G), respectively, are purely a result of rescaling the *y*-axis. The colours in (A) represent different cultivars as presented in Fig. 1 and the different colours in (D) to (G) represent an increased level of resistance from green to black.

The model is used to study the adaptation of lesion size in an agricultural landscape with both a susceptible and a quantitatively resistant host cultivar. However, let us first consider the case of a homogeneous landscape containing only plants of the susceptible cultivar. In this case, we find that the lesion size adapts towards an optimum value and will not continually increase. This is because the relationship between spore production per lesion and lesion size is bounded for large lesion sizes. As illustrated in Fig. 2, this results in a maximum for the spore production capacity (spores per mm^2^ lesion area). In a homogeneous landscape containing only one cultivar, the pathogen fitness is directly related to the spore production capacity, that is, the trait targeted by the resistance QTL (note that this is not necessarily the case when both cultivars are present in the landscape). Therefore, as in our evolutionary analysis we attempt to maximise pathogen fitness, the maximum spore production capacity then determines the evolutionary output of the PIP analysis (and the optimum lesion size).

**Figure 3:**
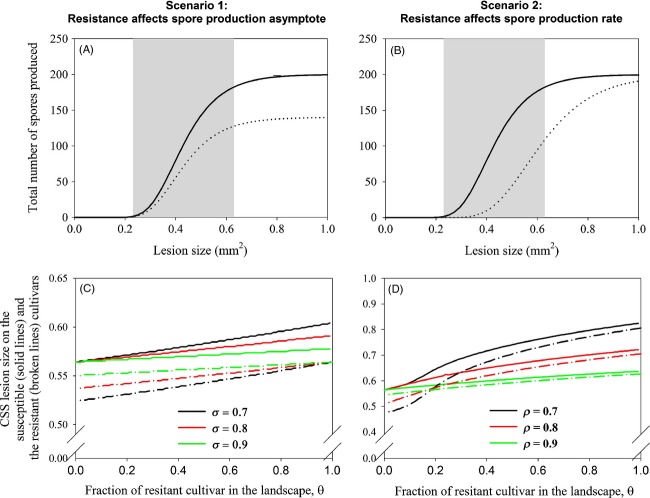
(A) and (B) Relationship between the number of spores produced per lesion and the lesion size for lesions growing on the susceptible (solid line) and resistant (dotted line) cultivar and their effects on and (C) and (D) the CSS lesion size for different cropping ratios, *θ*, and different levels of resistance (*σ* and *ρ*, respectively). Quantitative resistance affects either the upper asymptote [(A) and (C)] or the slope [(B) and (D)] of the spore production per lesion relationship. Note that it is assumed that lesions of the same isolates are generally smaller on the resistant cultivar as compared with the susceptible cultivar. The shaded areas represent the lesion size range found within the data set.

Figures 3C and d reveal how the introduction of a quantitatively resistant cultivar affects the lesion size adaptation and consequently the optimum (CSS) lesion size for a landscape containing a certain proportion of the resistant cultivar. The principal results are summarised in Table 2.

**Table 2:** Summary of principal results with respect to the CSS (continuously stable strategy) lesion size and total healthy host density, in landscapes with both susceptible, *S*, and quantitatively resistant, *R*, cultivars of wheat. The fraction of resistance within the landscape is denoted by *θ*.

	Resistance scenario 1: resistance affects upper limit of spore production with respect to lesion size (*cf*. Fig. 3A,C)	Resistance scenario 2: resistance affects growth rate of spore production with respect to lesion size (*cf*. Fig. 3B,D)
*CSS lesion sizes (LS)*		
*θ * ≈ 0; ∼*S* only	*LS* on *S *≈ *LS* in homogeneous *S* landscape	*LS* on *S *≈ *LS* in homogeneous *S* landscape
	*LS* on *R *≪ *LS* in homogeneous *S* landscape	*LS* on *R *≪ *LS* in homogeneous *S* landscape
Heterogeneous landscape containing *S* and *R*	Linear increase in *LS* on *S*&*R* as fraction of *R* increases in the landscape	Nonlinear increase in *LS* on *S*&*R* as fraction of *R* increases in the landscape
*θ *≈ 1; ∼*R* only	*LS* on *R *≈ *LS* in homogeneous *R* landscape	*LS* on *R *≈ *LS* in homogeneous *R* landscape
	*LS* on *S *≫ *LS* in homogeneous *R* landscape	*LS* on *S *> *LS* in homogeneous *R* landscape

In the case of resistance scenario 1, whereby resistance affects the upper limit of the relationship between lesion size and spore production per lesion (Fig. 3A), the introduction of the resistant cultivar within the landscape leads to a progressive increase in the CSS lesion size of the pathogen. When θ ≈ 0 (i.e. mainly plants of the susceptible cultivar), the CSS lesion size on the susceptible plants is close to the CSS lesion size in a homogeneous landscape containing only the susceptible cultivar. Increasing the frequency of resistant plants imposes a selection pressure and the CSS lesion size increases on both the resistant and the susceptible cultivar (Fig. 3C). Note that the lesion size on resistant plants remains smaller than on susceptible because of the assumption that lesions are in general smaller on the resistant cultivar. When *θ *≈ 1 (i.e. mainly plants of the resistant cultivar), the CSS lesion size on the resistant plants is close to the CSS lesion size in a homogeneously resistant landscape and the CSS lesion size on the susceptible plants has increased accordingly (Fig. 3C). These effects are enhanced by the strength of the resistance as determined by parameter *σ* (see coloured lines in Fig. 3C. Note that in this scenario, resistance affects only the upper limit of the relationship between spore production per lesion and lesion size (Fig. 2D). A consequence is that the lesion size at which the maximum spore production capacity is reached is not affected by the strength of the resistance *σ* (Fig. 2E). This explains that in a homogeneous landscape containing only the resistant cultivar (*θ *≈ 1), the CSS lesion sizes are equal for all values of *σ* (Fig. 3C). In the absence of a general fitness penalty (i.e. the lesions are not generally smaller on the resistant cultivar), the results are qualitatively the same (results not shown).

For resistance scenario 2, whereby the resistance affects the slope of the relationship between spore production per lesion and lesion size, the results are similar in that there is again a progressive increase in the CSS lesion size on both the susceptible and the qualitatively resistant cultivar as the fraction of the resistant cultivar within the landscape increases (Fig. 3D). However, contrary to scenario 1, the difference in lesion sizes between the resistant and the susceptible cultivars decreases as the fraction of the resistant cultivar within the landscape increases. This is because of the difference in the relationship between spore production and lesion size in scenarios 1 and 2, especially for high lesion size values (compare Fig. 3A,B). This is also because in scenario 2 the lesion size at which the maximum spore production capacity is reached is affected by the strength of the resistance, *ρ*, (Fig. 2E). Consequently, in a homogeneous landscape containing only the resistant cultivar (*θ *= 1), the CSS lesion sizes differ for different values of *ρ* (Fig. 3D). In the absence of a general fitness penalty (i.e. the lesions are not generally smaller on the resistant cultivar), adaptation towards increased lesion sizes does not occur (results not shown).

## Discussion

The idea that quantitative traits of the host–pathogen interaction can be under independent genetic control is sustained by many studies showing variety by isolate interactions for such traits (see Pariaud et al. (2009a,b2009b) for a review) and is reinforced by recent studies on the genetic support of quantitative resistance (Chung et al. 2010). In a paper on *Puccinia triticina* adaptation to wheat, Pariaud et al. (2009a,b2009b) decomposed the spore production per lesion (micrograms of spores) into lesion size (the size of a uredinium, in mm^2^) and spore production capacity, defined as the amount of spores produced per unit lesion area (micrograms of spores/mm^2^). They compared three *P. triticina* pathotypes (P1, P2 and P3) for these traits on a wheat variety and found that they presented different pathogenicity profiles: P2 produced large lesions but had a low spore production capacity, which suggests a good ability for growth within host tissues but a poor ability for host resource exploitation for spore production. P3 presented the opposite profile, with small lesions but a high spore production capacity, and P1 presented high values for both traits. The authors concluded that lesions size and spore production capacity are under independent genetic control and are likely to evolve independently. In a study on *Phytophthora infestans*, Carlisle et al. (2002) measured several traits of the host–pathogen interaction on three varieties of potato. They found that the lesion expansion rate was significantly correlated with the latent period but not with the spore production capacity. Their data clearly show isolates with a low spore production capacity and a high lesion growth rate, and reciprocally.

**Figure 4:**
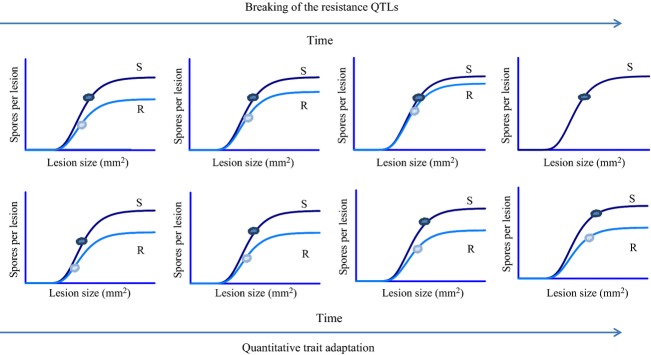
Graphical representation of the consequences for pathogen evolution of deploying quantitative resistance. Increased pathogen fitness measured by the composite trait ‘spores produced per lesion’ may be achieved in two ways: increasing the lesion's spore production capacity (the amount of spores produced per mm^2^ lesion surface) or increasing the lesion size. When the pathogen increases its spore production capacity by overcoming the associated resistance QTLs (top panels), this results in an increased number of spores produced per lesion on plants of the resistant cultivar only. However, when the pathogen tries to increase its fitness when confronted with a quantitatively resistant cultivar by increasing the magnitude of a trait that is not targeted by the QTL, e.g. lesion size (lower panels) this goes paired with an indirect effect on the composite trait (i.e. the spore production per lesion) which results in an increased lesion size on the plants of both cultivars within the landscape. The cloud shapes represent the most frequent pathogen isolates. Note that in addition to the specific resistance QTL affecting only the spore production capacity, we include a general resistance penalty resulting in lesions to be generally smaller on the more resistant cultivar.

There are, however, still few data available regarding the genetic support of the quantitative traits of the host–pathogen interaction. Most QTL studies with regard to quantitative resistance are based either on a global measurement of disease severity or on the measurement of a single trait. Resistance QTL studies for foliar diseases nevertheless suggest that distinct mechanisms govern different macroscopic components of resistance, such as lesion formation, lesion expansion or incubation period (Chung et al. 2010). In the case of *P. triticina*, Azzimonti et al. (2013) detected variety x isolate interactions for several quantitative traits, including lesion size and spore production capacity and they identified QTLs that are specifically linked to these traits (Azzimonti 2012). In maize northern leaf blight, Chung et al. (2010) tested and confirmed the hypothesis that individual QTLs affect distinct stages of the pathogen development. Another example of resistance QTLs having a specific effect on a pathogen quantitative trait can be found in Jorge et al. (2005). On the other hand, quantitative resistance has sometimes been found to pleiotropically affect pathogen development (e.g. Lehman and Shaner 1997) and it has been hypothesised that it could be assimilated to a form of nonhost basal resistance (González et al. 2012). These views are not necessarily in contradiction and it is reasonable to assume that a diversity of situations exists, with QTLs affecting either large parts of the pathogen development or a single epidemic trait. The present paper deals more specifically with the second case. We however did not ignore the possibility of a larger effect of the resistance QTL by including a basal reduction in lesion size for the resistant pathogen in the model (see Fig 4). Our main hypothesis remains that the pathogen lesion size is still able to evolve in the presence of a QTL that mainly affects another trait.

The data analysis revealed that, for *P. triticina*, spore production per lesion is positively correlated with lesion size and that the relationship differs amongst cultivars (*cf*. Table S2). Further data analysis revealed that isolates lie in a fixed order across the cultivar-specific regression lines in the sense that infections by an isolate that result in small lesions on a susceptible cultivar generally also result in relatively small lesions upon infection of a more resistant cultivar (Fig. 2; Table S3). This suggests that lesion size is at least partly determined by the isolate genotype and that when it increases on one cultivar, it systematically increases on other cultivars as well. As lesion size and spore production per lesion are correlated on each cultivar, increasing the lesion size also leads to an increase in the spore production per lesion on both the resistant and susceptible cultivars. This shows that the pathogen can indeed increase its spore production per lesion and hence its fitness through the adaptation towards increased lesion sizes. The isolates on cultivar *The* do not all follow the same general trend, as on the other cultivars. A possible explanation could be the presence of isolate-specific QTLs in this cultivar (or host-specific QTLs for pathogenicity in the isolates), resulting in an isolate–cultivar interaction for the measured quantitative trait. Such isolate-specific QTLs have been found in several host–pathogen systems (González et al. 2012). The data analysis therefore indicates that spore production per lesion is an increasing function of lesion size and depends both on the host (differences in slopes) and the pathogen (ranking of the isolates). The differences in the slopes can be interpreted as differences in spore production capacities accounted for by the host. Such differences were not tested for the pathogen, because such tests fall outside the scope of the current paper. However, for a more complete analysis of spore production capacity and lesion size with regard to host and pathogen genotypes, see Azzimonti (2012) and Azzimonti et al. (2013).

In this study, we assumed that the spore production capacity of the pathogen, expressed as the amount of spores it can produce per unit of sporulating tissue, is limited by the action of a resistance QTL in the host. In the classical studies into the evolutionary consequences of the introduction of quantitative resistance into the landscape, it is considered that the pathogen might overcome the resistance QTLs. Our simulations differ from this classical approach in that the resistance QTL remains effective throughout the simulations (parameters *σ* and *ρ* are kept constant). Despite this limitation, the pathogen is able to increase the number of spores it produces in a lesion and therewith to restore its transmission capacity, through the selection of strains with larger lesion sizes. Figure 4 illustrates the two distinct pathways to increased pathogen fitness after the introduction of quantitative plant resistance. The top row shows the gradual overcoming of a quantitative resistance that limits the spore production capacity in the pathogen: the spore production on the resistant host gradually increases to reach that on the susceptible host. The bottom row shows what happened in the simulations: the selection operating on the lesion size allows the pathogen to restore a high spore production per lesion on the resistant hosts, but this simultaneously affects the susceptible hosts.

Normally, it is assumed that when the healthy host density of the quantitatively resistant cultivar is starting to decline, the resistance is broken (i.e. the pathogen overcomes the resistance QTL), but in this paper, we have shown that this is not necessarily the case and that the observed increase in disease severity could instead be due to the selection of increased pathogenicity through selection on a pathogen trait that is not affected by the resistance QTLs. In such a case, both the resistant and susceptible cultivar are affected (Fig. 4). When monitoring the effects of the introduction of quantitative resistance in the field, it is thus essential to not merely compare the healthy host densities of the resistant cultivar to those of its susceptible counterpart, because in the case of quantitative trait adaptations, the difference in disease severity between the cultivars might not have been significantly affected, although there might be a significant absolute increase in the disease severity on both cultivars.

This study is based on the relationship between two quantitative traits of the host–pathogen interaction. Although published data on this question are still limited, such relationships, positive or negative, can be found amongst other traits. For example, Pariaud et al. (2012) have established a positive link between the duration of the latent period and the spore production capacity of wheat leaf rust, resulting in an evolutionary trade-off. The question whether the use of quantitative resistance affecting the spore production could lead to the selection of pathogens with shorter latent periods would thus deserve further attention. In future, studies on pathogen adaptation to quantitative resistance should account better for the existence of cultivar-specific relationships between quantitative traits.

A general concern in plant pathology has been that the presence of quantitative resistance selects for an increased pathogenicity as accounted for by quantitative traits (Garrett and Mundt 1999; Mundt 2002). Our analysis reveals that this concern is well founded and should be taken into account in resistance management strategies. A similar conclusion is reached by Gandon and Michalakis (2000) with a different approach. They compared the evolution of parasite-induced host death under selection by qualitative or quantitative host resistance. Their main prediction is that, by overcoming the host quantitative resistance, the parasite will increase its capacity to damage the host on both the resistant and susceptible hosts. A main assumption of this model is however that quantitative resistance is a way for the host to limit the deleterious effects induced by the parasite but that it does not act directly on transmission. Applying their model to plant pathogens is then not straightforward because quantitative resistance often also affects the transmission capacity (spore production) of the pathogen. With an approach based on an alternative hypothesis, we have extended the scope of Gandon and Michalakis (2000) their predictions to plant foliar pathogens.

## Data archiving statement

The raw experimental data and additional analyses are available as electronic supplementary materials attached to the online version of the publication.

## Supporting Information

**Data S1.** Raw experimental data. **Table S1.** Overview of isolates and cultivars used for the regression analysis. **Table S2.** Regression analysis on the total spore weight produced per lesion (*S_p_* in mg) as a function of the sporulating lesion area (*A*, in cm2) with a cultivar (*C_v_*) grouping factor. **Table S3.** Spearman ranking correlation coefficients for isolates 2, 7, 8, 9, 11 and 12 as listed in Fig. 2. **Table S4.** Spearman ranking correlation coefficients for isolates 1, 3, 4, 5, 6 and 10 as listed in Fig. 2. **Table S5.** Spearman ranking correlation coefficients for isolates 2, 7, 8, 9, 11 and 12 as listed in Fig. S1. **Table S6.** Spearman ranking correlation coefficients for isolates 1, 3, 4, 5, 6 and 10 as listed in Fig. S1. **Figure S1.** Spore production and lesion size relationship for (a) the raw data and (b) the ranking of the transposed medians across the estimated cultivar regression lines with the cultivar Morocco as the reference cultivar.
