# Supplementary material for: Quantitative resistance can lead to evolutionary changes in traits not targeted by the resistance QTLs
Source: Evol Appl. 2014 Jan 2;7(3):370–80. doi: 10.1111/eva.12130 (PMC3962297; doi:10.1111/eva.12130)
Supplement: Supplementary file 2 [file eva0007-0370-sd2.docx]

**Supplementary materials**

**Summary of data available for regression analysis**

An existing data set was used to determine whether there is a cultivar specific relationship between spore production per lesion and lesion size. These data were collected during an experiment designed to measure pathotype and isolate differences in aggressiveness components and adaptation to a range of host cultivars for the wheat leaf rust *Puccinia triticina* (See Pariaud ([2008](#_ENREF_3)) and Pariaud et al. ([2009](#_ENREF_4)) for experimental procedures). Table S1 summarises the host cultivars and *P triticina* isolates for which lesion size and spore weight per lesion measurements were available.

**Mathematical details for evolutionary dynamics modelling**

The evolutionary dynamics of the system are analysed with the method of pair-wise invasibility plots (PIPs), a technique borrowed from adaptive dynamics ([Maynard Smith 1982](#_ENREF_2); [Geritz et al. 1998](#_ENREF_1)). To construct the PIPs we numerically determine whether a mutant that has a slightly altered lesion size and thus a slightly altered spore production capacity can invade the resident pathogen system when at equilibrium, whereby invasion success is determined by the invasion criterion. Linearising equations (2) about its steady state solutions and adding a small quantity of invader yields the Jacobian, *J*, of the system. The dominant eigenvalue, λ, of $\det(J-\lambda I)=0$ represents the growth factor of the invader population in the host mixture infected by the resident pathogen and therewith denotes the invasion criterion. The PIPS are subsequently calculated numerically from the invasion criterion $\lambda=0$ and show for each resident lesion size value, $LS_{S r}$, all invader lesion size values, $LS_{S i}$, for which the initially rare invader, in the environment set by the resident, either invades the system and replaces the resident population (the mutant has a positive global population growth rate; $\lambda>0$) or dies out (the mutant has a negative global population growth rate; $\lambda<0$). Evolutionary isoclines separate areas of successful invasion from those of unsuccessful invasion. Since the resident phenotype value is neutral in its own environment the evolutionary isoclines always include the main diagonal, i.e. for $LS_{S i}=LS_{S r}$ the mutant’s global population growth rate is zero. Lesion sizes for which the evolutionary isoclines intersect are known as evolutionarily singular strategies and denote potential evolutionary endpoints. The singular strategies can subsequently be classified visually from the PIP as expounded in Geritz *et al.* ([1998](#_ENREF_1)). All singular strategies presented in this paper are both evolutionary stable, whereby evolution is towards the singular strategy and once reached cannot be invaded by invaders with similar strategies, and convergence stable and will thus be referred to as continuously stable strategies or CSSs. Phenotypic changes are assumed to be small and to occur rarely enough for the system to have converged to equilibrium before the next mutation occurs. Note that the lesion size on the resistant cultivar can be derived from equation (6) or (7).

**Detailed regression analysis results**

The regression analysis revealed that the full model as defined by equation (1) accounted for 74.3% of the variance in the data and identified a monotonically rising relationship between lesion size and spore production per lesion ($p<0.001$). Moreover, the regression lines were shown to be cultivar specific with significantly different intercepts ($p<0.001$) and slopes (; Table S2).

**Table S1.** Overview of isolates and cultivars used for the regression analysis.

| Isolate | Reference no. | Total number of replicates per cultivar | | | | |
| --- | --- | --- | --- | --- | --- | --- |
|  |  | Morocco (Mor) | Soissons (Soi) | Scipion (Scp) | Festival (Fes) | Thésée (The) |
| 7-09 | 1 | 5 | 5 |  |  | 5 |
| 0-032 | 2 | 5 | 5 | 5 | 5 |  |
| 9-78 | 3 | 5 | 5 |  |  | 5 |
| 9-170 | 4 | 5 | 5 |  |  | 5 |
| Ob6 | 5 | 5 | 5 |  |  | 5 |
| Cd0d3 | 6 | 4 | 5 |  |  | 5 |
| 9-44 | 7 | 5 | 5 | 5 | 5 |  |
| 1-026 | 8 | 5 | 5 | 5 | 5 |  |
| 1-073 | 9 | 5 | 5 | 4 | 5 |  |
| 9-51 | 10 | 5 | 5 |  |  | 5 |
| 1-257 | 11 | 5 | 5 | 5 | 4 |  |
| 1-096 | 12 | 5 | 5 | 5 | 5 |  |

**Table S2.** Regression analysis on the total spore weight produced per lesion (*Sp* in mg) as a function of the sporulating lesion area (*A*, in cm^2^) with a cultivar (*Cv*) grouping factor.

| **Effect** | **df** | **Mean square** | **Variance ratio** | **F pr.** |
| --- | --- | --- | --- | --- |
| *A* | 1 | 1.951377 | 451.04 | <.001 |
| *Cv* | 4 | 0.156403 | 36.15 | <.001 |
| *A.Cv* | 4 | 0.010786 | 2.49 | 0.044 |
| Residual | 197 | 0.004326 |  |  |
| Total | 206 | 0.016856 |  |  |

**Detailed spearman ranking correlations results for isolate means**

A spearman ranking coefficient test of the ranks of the isolate means along the cultivar specific regression lines was used to test whether the isolates lie across these regression lines in a fixed order. Cultivars *Mor*, *Soi*, *Fes* and *Scp* have a total of six isolates in common (*cf.* Table S1). For these data the spearman ranking coefficient test revealed that there is a strongly significant correlation between the order of the isolate means on the different regression lines (Table S3). Cultivars *Mor*, *Soi* and *The* also have six isolates in common and although the order of the isolate means is significantly similar for *Mor* and *Soi* the isolate means lie along the *The* regression line in a different order (Table S4). Cultivar *Soi* and *Mor* have all twelve isolates in common. When all twelve isolates were taken into account the analysis still found a strongly significant correlation between the order of the isolate means along the estimated regression lines for these two cultivars ($\rho=0.902;p<0.001$).

**Detailed spearman ranking correlations results for isolate medians**

Additional analysis was performed to investigate how the isolate ranking results are affected when the isolates are ranked according to their median values rather than their mean. Figure S1 shows the orientation of the projected (according to their perpendicular) median isolate values across the cultivar specific regression lines, which represent the relationship between lesion size and spore production per lesion. A spearman ranking coefficient test of the order of the isolate medians across the individual cultivar specific estimated regression lines for cultivars *Mor*, *Soi*, *Fes* and *Scp*, which have a total of six isolates in common, reveals that as for the mean based rankings, there is a significant correlation between the order of the isolate medians for these four cultivars (Table S5). Cultivars *Mor*, *Soi* and *The* also have six isolates in common. When isolates are ranked according to their median the order of the medians is significantly similar for *Mor* & *Soi* and *Soi* & *The*, but not for *Mor* & *The* (Table S6). This only slightly differs from the results based on the rankings according to isolate means rather than medians, which found that the order of the isolate means along the regression lines was not significantly correlated for both *Soi* & *The* and *Mor* & *The* (Table S4).

A direct comparison of the isolate order derived according to mean values and the isolate order derived according to median values for cultivar *Mor* (our reference cultivar) reveals that the rankings for both methods of averaging the isolate values are significantly correlated ($\rho=0.986;p<0.001$). This shows that the order is not significantly affected by the method of averaging. In conclusion, the qualitative results seem unaffected by whether the isolate rankings across the cultivar specific regression lines are determined according to isolate mean or isolate median values.

**Table S3.** Spearman ranking correlation coefficients for isolates 2, 7, 8, 9, 11 and 12 as listed in Fig. 2. The associated probabilities are given in brackets (*cf.* Fig. 2 and Table S1).

| **Mor** | **1** | 1.000 |  |  |  |
| --- | --- | --- | --- | --- | --- |
| **Soi** | **2** | 0.943 (0.001) | 1.000 |  |  |
| **Fes** | **3** | 0.943 (0.001) | 1.000 (0.000) | 1.000 |  |
| **Scp** | **4** | 0.943 (0.001) | 0.829 (0.008) | 0.829 (0.008) | 1.000 |
|  |  | **1** | **2** | **3** | **4** |

**Table S4.** Spearman ranking correlation coefficients for isolates 1, 3, 4, 5, 6 and 10 as listed in Fig. 2. The associated probabilities are given in brackets (*cf.* Fig. 2 and Table S1).

| **Mor** | **1** | 1.000 |  |  |
| --- | --- | --- | --- | --- |
| **Soi** | **2** | 0.886 (0.004) | 1.000 |  |
| **The** | **3** | 0.143 (0.178) | 0.314 (0.124) | 1.000 |
|  |  | **1** | **2** | **3** |

**Table S5.** Spearman ranking correlation coefficients for isolates 2, 7, 8, 9, 11 and 12 as listed in Fig. S1. The associated probabilities are given in brackets (*cf.* Fig. S1 and Table S1).

| **Mor** | **1** | 1.000 |  |  |  |
| --- | --- | --- | --- | --- | --- |
| **Soi** | **2** | 0.943 (0.001) | 1.000 |  |  |
| **Fes** | **3** | 1.000 (0.000) | 0.943 (0.001) | 1.000 |  |
| **Scp** | **4** | 0.829 (0.008) | 0.771 (0.015) | 0.829 (0.008) | 1.000 |
|  |  | **1** | **2** | **3** | **4** |

**Table S6.** Spearman ranking correlation coefficients for isolates 1, 3, 4, 5, 6 and 10 as listed in Fig. S1. The associated probabilities are given in brackets (*cf.* Fig. S1 and Table S1).

| **Mor** | **1** | 1.000 |  |  |
| --- | --- | --- | --- | --- |
| **Soi** | **2** | 0.600 (0.044) | 1.000 |  |
| **The** | **3** | 0.257 (0.141) | 0.829 (0.008) | 1.000 |
|  |  | **1** | **2** | **3** |

**Figure S1.** Spore production and lesion size relationship for (a) the raw data and (b) the ranking of the transposed medians across the estimated cultivar regression lines with the cultivar Morocco as the reference cultivar. The numbers in both graphs refer to the isolates as listed in Table S1.

**Literature cited**

Geritz, S. H. A., E. Kisdi, G. Meszena, and J. A. J. Metz. 1998. Evolutionary singular strategies and the adaptive growth and branching of the evolutionary tree. *Evolutionary Ecology* 12:35-57.

Maynard Smith, J. 1982. *Evolution and the Theory of Games*. Cambridge: Cambridge University Press.

Pariaud, B. 2008. Agressivité de *Puccinia* *triticina* (agent de la rouille brune du blé) et son adaptation à l'hôte - Aggressiveness of *Puccinia* *triticina* (causing wheat leaf rust) and adaptation to its host, Paris XI University.

Pariaud, B., C. Robert, H. Goyeau, and C. Lannou. 2009. Aggressiveness components and adaptation to a host cultivar in wheat leaf rust. *Phytopathology* 99:869-878.
